# Effect of intraoperative paravertebral or intravenous lidocaine versus control during lung resection surgery on postoperative complications: A randomized controlled trial

**DOI:** 10.1186/s13063-019-3677-9

**Published:** 2019-11-06

**Authors:** Francisco De la Gala, Patricia Piñeiro, Almudena Reyes, Carlos Simón, Elena Vara, Lisa Rancan, Luis Javier Huerta, Guillermo Gonzalez, Carmen Benito, Marta Muñoz, Pilar Grande, Sergio D. Paredes, Pablo Tomas Aznar, Alvaro Perez, David Martinez, Fernando Higuero, David Sanz, Juan Pedro De Miguel, Patricia Cruz, Luis Olmedilla, Elena Lopez Gil, Patricia Duque, Guillermo Sanchez-Pedrosa, Mayte Valle, Ignacio Garutti

**Affiliations:** 1 Department Anesthesiology, Hospital General Universitario Gregorio Marañón, Madrid, Spain; 2 Department Thoracic Surgery, Hospital General Universitario Gregorio Marañón, Madrid, Spain; 3 Biochemical Department, School of Medicine, Universidad Complutense de Madrid, Madrid, Spain

**Keywords:** Lidocaine, Lung resection surgery, Postoperative pulmonary complications, Postoperative complications

## Abstract

**Background:**

Use of minimally invasive surgical techniques for lung resection surgery (LRS), such as video-assisted thoracoscopy (VATS), has increased in recent years. However, there is little information about the best anesthetic technique in this context. This surgical approach is associated with a lower intensity of postoperative pain, and its use has been proposed in programs for enhanced recovery after surgery (ERAS). This study compares the severity of postoperative complications in patients undergoing LRS who have received lidocaine intraoperatively either intravenously or via paravertebral administration versus saline.

**Methods/design:**

We will conduct a single-center randomized controlled trial involving 153 patients undergoing LRS through a thoracoscopic approach. The patients will be randomly assigned to one of the following study groups: intravenous lidocaine with more paravertebral thoracic (PVT) saline, PVT lidocaine with more intravenous saline, or intravenous remifentanil with more PVT saline. The primary outcome will be the comparison of the postoperative course through Clavien-Dindo classification. Furthermore, we will compare the perioperative pulmonary and systemic inflammatory response by monitoring biomarkers in the bronchoalveolar lavage fluid and blood, as well as postoperative analgesic consumption between the three groups of patients. We will use an ANOVA to compare quantitative variables and a chi-squared test to compare qualitative variables.

**Discussion:**

The development of less invasive surgical techniques means that anesthesiologists must adapt their perioperative management protocols and look for anesthetic techniques that provide good analgesic quality and allow rapid rehabilitation of the patient, as proposed in the ERAS protocols. The administration of a continuous infusion of intravenous lidocaine has proven to be useful and safe for the management of other types of surgery, as demonstrated in colorectal cancer. We want to know whether the continuous administration of lidocaine by a paravertebral route can be substituted with the intravenous administration of this local anesthetic in a safe and effective way while avoiding the risks inherent in the use of regional anesthetic techniques. In this way, this technique could be used in a safe and effective way in ERAS programs for pulmonary resection.

**Trial registration:**

EudraCT, 2016–004271-52; ClinicalTrials.gov, NCT03905837. Protocol number IGGFGG-2016 version 4.0, 27th April 2017.

**Electronic supplementary material:**

The online version of this article (10.1186/s13063-019-3677-9) contains supplementary material, which is available to authorized users.

## Background

Surgical stress triggers a local and systemic inflammatory response, with cytokine expression modulating the inflammatory process. An exaggerated perioperative inflammatory response during lung resection surgery (LRS) has been associated with the appearance of postoperative complications [1, 2]. An association has also been demonstrated between inflammation and postoperative kidney damage [3], supraventricular tachyarrhythmias [4], postoperative cognitive dysfunction [5], and the intensity of peri-operative pain [6]. LRS by video-assisted thoracic surgery (VATS) was shown to provide improvements for the patient with regard to both pain management and quality of life compared to thoracotomy [7] and open lung resection. However, there is less information about what the best type of analgesia is when performing pulmonary resection by VATS.

Thoracic paravertebral block (TPVB) is a peripheral nerve block that has similar effectiveness to thoracic epidural anesthesia (TEA) for the management of thoracotomy pain and is also associated with a lower incidence of complications [8]. Its use is widely accepted in open thoracic surgery. However, there is less information about its use in LRS-VATS. A recent meta-analysis showed that TPVB in VATS is associated with better quality of pain management than a control intervention. It also reduces the consumption of postoperative anesthesia for 48 h and the length of hospital stay [9]. This regional technique is associated with an attenuation of the perioperative response to stress according to measurements of inflammatory biomarkers [10, 11] and stress hormones [12].

Lidocaine is the only local anesthetic (LA) that is safe for intravenous (IV) use. We have known for years that IV lidocaine has systemic analgesic effects, and its intravenous administration intraoperatively decreases opioid consumption. Anti-inflammatory effects associated with the use of local anesthetics have also been described [13]. The use of a continuous IV infusion of lidocaine during surgery is associated with a lower systemic inflammatory response according to plasma cytokine levels, as well as less postoperative pain, a shorter duration of postoperative paralytic ileus, and earlier hospital discharge [14–17]. At the experimental level, our group demonstrated that administration of IV lidocaine in pigs subjected to LRS (lobectomy) decreased proinflammatory cytokine expression in the liver and bronchoalveolar lavage (BAL) fluid, and it also decreased pulmonary edema [18, 19]. These effects were observed both intraoperatively and at 24 h postoperatively. We hypothesized that IV or paravertebral (PV) administration of lidocaine would attenuate the systemic and pulmonary inflammatory response that patients usually develop, which would result in a better postoperative course.

## Methods/design

### Methods

#### Study design and setting

This study will be conducted at a single center (an academic hospital) as a randomized, double-blind, controlled, phase IV clinical trial with three parallel groups. It will be performed in accordance with the legislation in Spain, Royal Decree 1090/2015 of December 4 and Law 14/2007 (Biomedical Research Law). The law requires carrying civil liability insurance that covers possible damages that may result for patients included in the study and guaranteeing compliance with data protection laws. The trial was approved by the clinical research ethics committee of Gregorio Marañón University Hospital on April 2018 and is registered with EudraCT (2016–004271-52) and ClinicalTrials.gov (NCT03905837).

#### Patients

The trial will include patients who are over 18 years old and are legally capable. Patients of both sexes who are scheduled to undergo LRS in the thoracic surgery department at Gregorio Marañón Hospital will be included. Participation will be voluntary and signed informed consent forms will be collected. Participants will be required to pass functional respiratory tests with forced expiratory volume at one second > 50% or forced vital capacity > 50% preoperatively, and they will not have received chronic treatment with oral corticosteroids or immunosuppressants 3 months before surgery. Participants must also have no previous history of liver disease. The exclusion criteria are pregnancy or lactation, known hypersensitivity to amide-type local anesthetics, transfusion of blood products in the past 10 days, and an inability to undergo mechanical ventilation for pulmonary protection.

#### Intervention plan

**Fig. 1:**
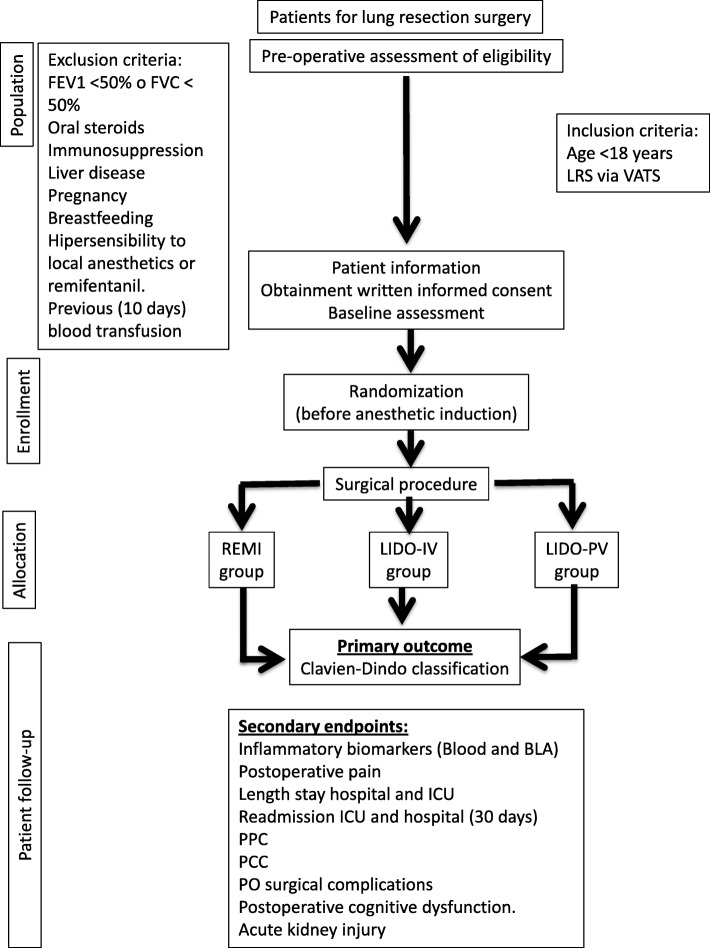
Consort list

The participant timeline is shown in Fig. 1. All of the patients included in the study will be managed with the same preoperative and postoperative protocols, which include antithrombotic and antibiotic prophylaxis, fasting to clear fluids until 2 h before the surgery, restrictive fluid therapy with crystalloids (less than 1.5 L of positive balance in the first 24 h), postoperative PV analgesia, pulmonary protection ventilation during the one-lung ventilation (OLV) period (tidal volume less than 6 mL/kg ideal weight), and an early mobilization of the patients. The acute pain unit (APU) at our center will manage the postoperative pain. The PV analgesic infusion will be stopped 48 h after the surgery.

At the preoperative visit, a member of the research team will visit the candidate to explain the study. If a patient agrees to be included in the study, they will be asked to sign an informed consent form and undergo a mini-mental state examination. No anxiolytics will be administered to the patients before the surgery.

### Randomization

Patients will be randomized into the following three groups (1:1:1 ratio): intravenous lidocaine and PV saline (group 1); IV and PV saline solution with lidocaine (group 2); or IV remifentanyl and PV saline solution (group 3). The randomization will be performed using the software EPIDAT 3.1 before the first patient is recruited, and the randomization codes will be kept in a sealed envelope with a number on the outside of the envelope.

A study nurse will be in charge of preparing the medication according to the result obtained in the envelope. The surgical anesthesiologist will receive the assigned and prepared medication for the corresponding group with only the identification of IV perfusion (500 mL) and PV perfusion (100 mL) without knowledge of the group to which the patient belongs. The content of solutions will be indistinguishable to the investigating team because all of the solutions are clear liquids.

For bias control, the physicians involved will be blinded to the techniques used, including the anesthesiologist responsible for the intraoperative management of the patient, the physician responsible for postoperative control in the postoperative care unit, and the guard physician who will perform the cognitive dysfunction test preoperatively and postoperatively, as well as the follow up until the month of surgery. Those responsible for analysis of the biological samples will also be blinded.

### Interventional treatment

Patients will be recruited consecutively and randomized into three groups of intraoperative management, as follows:

- Group 1: Experimental arm—intravenous lidocaine and PV saline (SS). During intraoperative anesthetic maintenance, a continuous IV infusion of lidocaine at 1.5 mg/kg/h will be administered until the end of surgery, and perfusion of 0.9% SS will be administered through an intraoperative PV catheter at a rate of 0.1 mL/kg/h.
- Group 2: Experimental arm—intravenous SS and PV lidocaine. During anesthesia maintenance, a continuous intravenous infusion of 0.9% SS and an infusion of 2% lidocaine will be administered through an intraoperative PV catheter at a rate of 0.1 mL/kg/h.
- Group 3: Active comparator—intravenous remifentanil and PV SS. A continuous intravenous infusion of remifentanil will be administered at a rate of 0.1 mg/kg/min during anesthesia maintenance until the end of surgery, and an infusion of 0.9% SS will be administered through an intraoperative PV catheter at a rate of 0.1 mL/kg/ h.

The administration of the study drugs will be stopped immediately in cases where the study participant shows relevant deterioration (e.g., severe hypotension refractory to bolus dose vasoconstrictor treatment, or sudden life-threatening arrhythmia).

### Induction and monitoring

Immediately before anesthetic induction, all patients will be monitored using electrocardiograms, pulse oximetry, and capnography. In addition, patients will be monitored using the bispectral index, cerebral oxygen saturation*,* invasive arterial pressure, and peripheral quantitative neuromuscular status. Regardless of the group assigned, all patients will be induced with 2 mg/kg of propofol, 3 μg/kg of fentanyl, and 0.6 mg/kg of rocuronium. Tracheal intubation will be performed with a double-lumen tube. Correct placement will be verified with a fiberoptic bronchoscope.

The patients will then be placed in the lateral decubitus position for the surgery. In this position, a PV catheter will be inserted into the hemithorax (level T5–T6) with a 3-mL initial test dose of 0.25% bupivacaine plus epinephrine (1:200.000), followed by continuous infusion of lidocaine or SS depending on the randomized group assignment. The assigned IV infusion treatment will be started at this time with PV infusion.

The parameters applied during ventilation will be different at three time points: 1) the baseline (BAS) parameters from orotracheal intubation until the initiation of OLV in two-lung ventilation (TLV) will be volume-controlled ventilation, a tidal volume (TV) of 8 mL/kg (ideal weight), positive end expiratory pressure (PEEP) of 5 cmH_2_O, FiO_2_ of 0.4–0.5, and respiratory rate to maintain an end-tidal carbon dioxide at 35 mmHg; 2) the values applied during OLV will be a TV of 6 mL/kg (ideal weight), ideal PEEP calculated as the one that provides the best lung compliance in the recruitment maneuver, permissive hypercapnia, and FiO_2_ of 0.6–1 to maintain SaO_2_ > 90%; and 3) for lung re-insufflation (END), the same ventilation parameters will be applied as in BAS. Recruitment maneuvers will be performed, and continuous positive airway pressure will be used in the nondependent lung when needed to resolve hypoxemia (SpO_2_ < 90%). Restrictive fluid therapy with crystalloids will be administered at 2 mL/kg/h to maintain diuresis > 0.5 mL/kg/h. A fluid bolus of 250 mL of crystalloids will be administered when diuresis is < 0.5 mL/kg/h.

When closure of the thoracic incision begins, all the intervention infusions will be stopped, and a PV bolus of 0.15 mL/kg of 0.2% ropivacaine will be administered. The radial artery will be catheterized in all cases using the Pro AQT sensor to monitor the cardiac index, stroke volume variation, stroke volume index, and invasive arterial pressure. These values will be recorded at baseline during TLV, at 30 min after initiation of OLV, and at the end of OLV. Depending on the data recorded, vasoactive drugs will be administered to ensure optimal hemodynamic parameters for the intrapulmonary shunt. The respiratory parameters recorded during surgery will be as follows: TLV at baseline, 30 min after initiation of OLV, and the end of OLV, FiO_2_, SpO_2_, PaCO_2_, TV, minute volume, respiratory rate, peak pressure, plateau pressure, mean pressure, end expiratory pressure, lung dynamic compliance (Cdyn), and driving pressure.

### Sample and measurement methods

BAL samples will be taken from the dependent lung 5 min before initiating OLV (BAS) and at the end of OLV (END) once TLV is established. Sampling will be performed using a 4.5-mm fiber-optic bronchoscope wedged into the selected segment of the bronchus of the left lower lobe and middle or right lower lobe with 100 mL of 0.9% saline solution in 25-mL aliquots to analyze inflammatory markers (interleukin (IL)-1, IL-2, IL-4, IL-6, IL-7, IL-8, IL-10, IL-12, tumor necrosis factor (TNF)-α, monocyte chemoattractant protein, and vascular endothelial growth factor). Arterial blood will be drawn to measure PaO_2_, SaO_2_, PaCO_2_, and the following inflammatory markers analyzed in BAL at four time points: baseline (before OLV), 30 min after initiation of OLV, the end of OLV, and 24 h after surgery. BAL and blood samples will be centrifuged, and the supernatant will be analyzed at a specialized laboratory. Biomarker concentrations will be analyzed using western blotting. The relationship between pro-inflammatory and anti-inflammatory markers is measured using the ratios of IL-6/IL-10 and TNF-α/IL-10.

### Postoperative management

After surgery, all patients will be admitted to the postoperative care unit (PACU), where the PV analgesic infusion of 0.2% ropivacaine (0.1 L/kg/h) will be initiated. This PV analgesia will be maintained during the first 48 h after surgery. In the PACU, pain severity will be assessed at rest and during coughing using a numeric rating scale (0 = no pain, 10 = the worst imaginable pain). Furthermore, all patients will be provided with a patient-controlled analgesia pump (PCA) for the self-administration of PV rescue boluses of this solution during the first 48 h postoperatively. If the visual analogic scale (VAS) score is higher than 5 points, an intravenous bolus of 1 mg of morphine will be administered until the VAS reaches a value of less than 5 points.

We will record the number of PV boluses required and administered through the PCA pump within the first 48 h of the postoperative period. We will also record the morphine consumption during this period. Patients will be discharged from the PACU the following morning, except if the responsible physician considers that they should continue to have continuous surveillance.

#### Outcomes

##### Primary outcome

The primary outcome is the proportion of patients included in the different scales of the Clavien–Dindo (C–D) classification [20] in the three groups of patients. The greater the intensity of the treatment that is necessary to correct any postoperative complication, the higher the C–D classification will be considered. Any deviation from a normal postoperative course will be classified into grade I, II (minor complications), IIIa, IIIb, IVa, IVb, and V (major complications) of this classification (Appendix 1).

##### Secondary outcomes

Secondary objectives including the following will be the comparison between three groups of patients with the following laboratory or clinical data:

Secondary clinical outcomes

- Postoperative pain intensity: Postoperative pain will be evaluated using the VAS at rest, during movement, and when coughing during the stay in the PACU and in the two days after. We will also record the doses of self-administered rescue analgesics by PCA pump during the first 48 h after surgery, as well as the cumulative morphine consumption in the first 48 h post-operation.
- Postoperative pulmonary complications (PPCs): PPCs will be classified using the definition applied in the ARISCAT study [21] (atelectasis, suspected pulmonary infection, respiratory failure, bronchospasm, aspiration pneumonitis, pleural effusion, and pneumothorax; Appendix 2). Because pneumothorax and pleural effusion are the usual outcomes after LRS in the nondependent lung, we will consider them to be complications only if they occur in the dependent lung. The time frame is up to 30 days after intervention.
- Postoperative cognitive dysfunction (POCD) is defined as the presence of a mini-mental test value less than 27 points at 3 days postoperative (in patients with a value of 30 points on preoperative mini-mental test) or if there is a drop of more than 3 points compared to the value obtained preoperatively (in patients with a mini-mental preoperative test value less than 30 points).
- The incidence of postoperative surgical complications, which is defined as the presence of any of the complications during the first 30 days postoperatively, as follows:
  - ◦ Wound infection: Infection of the incisional surgical site, purulent drainage and/or isolation of pathogenic microorganisms
  - ◦ Prolonged air leak: Air escape through the thoracic drainage tubes beyond postoperative day 5
  - ◦ Bleeding: A surgical wound that requires re-intervention or the patient requires transfusion of blood products
  - ◦ Subcutaneous emphysema: Palpation of subcutaneous crepitation or the presence of subcutaneous air in a thoracic image (chest X-ray or computed tomography)
  - ◦ Bronchopleural fistula: Communication of the bronchus with the pleural space as shown on a thoracic image
  - ◦ Pleural empyema: Pleural effusion with the macroscopic presence of pus, a positive Gram stain or culture of pleural fluid, or a pleural fluid pH under 7.2 with normal peripheral blood pH
  - ◦ Cardiac herniation: Presence of the heart outside its expected position, as shown on a thoracic image
  - ◦ Surgical re-intervention: Patient who requires reoperation for any reason (pulmonary or not)
- Incidence of postoperative cardiac complications within 30 days postoperatively, if any of the following cardiac events occur:
  - ◦ Cardiac arrhythmias: defined as evidence of an abnormal heart rhythm electrocardiograph (ECG) that was not present before the intervention
  - ◦ Stroke episodes: defined as an acute focal injury of the central nervous system that has a vascular cause (embolic, thrombotic or hemorrhagic) with residual motor deficit, sensory, or cognitive dysfunction
  - ◦ Cardiac failure: The presence of new respiratory distress, S3, jugular venous distension, and a new chest X-ray finding of pulmonary vascular redistribution or interstitial
  - ◦ Myocardial ischemia: An increase in ultrasensitive troponin with at least one value above the 99th percentile of the upper reference limit and at least one of the following criteria: ECG changes compared to preoperative ECG that suggests ischemia or new echocardiographic (wall motion) abnormalities
  - ◦ Cardiac arrest
- Incidence of postoperative renal complications.
  Postoperative acute kidney failure is defined as an increase of serum creatinine more than 1.5 times or ≥ 0.3 mg/dL from baseline or urine output < 0.5 mL/kg/h for 6 h using the acute kidney injury network (AKIN) classification [22]. Time frame: up to 30 days after intervention.
- Length of stay in the hospital and PACU
- Unplanned readmissions to the hospital and PACU during the first 30 postoperative days

Secondary laboratory outcomes:

- Systemic inflammatory biomarkers (IL-1, IL-2, IL-6, IL-8, IL-10, TNF-α, and monocyte chemoattractant protein) in serum and BAL fluid. Neuroinflammatory markers (S-100 beta protein, neuronal specific enolase, and glial fibrillary acidic protein) measured in serum during the first 24 h postoperatively
- The gas exchange: measured as PaO_2_/FiO_2_ at 24 h after the intervention

### Adjudicating outcome variables

All the physicians who evaluate the clinical and laboratory postoperative course will be blinded to the group allocation. Postoperative pain will be evaluated by APU physicians. The presence of postoperative complications and the C–D classification will be assessed separately by two teams of investigators (thoracic surgeons and anesthesiologists) at hospital discharge. In cases of discrepancy, there will be a meeting between both teams to discuss the results and reach an agreement. In this meeting between researchers, the complications collected will be assessed to determine if they meet the criteria for their diagnosis, and those that meet the definition criteria will be recorded as a complication while those that do not meet the definition criteria will not be evaluated.

Length of stay in the hospital and PACU, readmission, and mortality at 30 days will also be recorded by the investigator team based on the hospital electronic register.

### Data collection and management

A data collection notebook (CRD) will be compiled with all the variables described. Patients will arrive at the operating room with the first sheet completed (including demographic, analytical, respiratory function, and preoperative morbidity information). During surgery, intraoperative information will be collected on the subsequent pages. Before registration, the CRD pages corresponding to the patient’s stay in the PCU will be filled out. The CRD information will be completed before discharge. The CRD will include the patient’s in-hospital medical history and information that is not computerized. At the time of discharge, it will be kept in a locked cabinet in the anesthesiology department and will be completed with the data obtained during the day-30 visit. Data from the CRDs will be entered into the computer program SPSS, where they will be reviewed and accepted by an external clinical research organization (CRO). This CRO will ensure compliance with the study procedure and will control the possible errors that may occur.

The criteria for withdrawal from the study are as follows:

- The protocol procedures cannot be followed for medical reasons
- Transfusion of intraoperative blood products
- Duration of OLV less than 60 min
- Adverse events related to any medication that is used during the study that prevents the continuation of the protocol
- The patient voluntarily decides to leave the study or withdraws informed consent
- Any adverse clinical event that requires withdrawal from the study in the opinion of the researcher
- Completion of the study

Patients who have to withdraw from the study because of these criteria will not be replaced by other patients. From the moment at which the withdrawal from the trial is decided, study samples of inflammatory markers will no longer be obtained. These patients will be followed by the members of the research team in the same way as patients who complete the study until hospital discharge, guaranteeing them the same healthcare. In these cases, only safety data (serious adverse events) will be collected for the study. For patients who withdraw their consent, no further patient data will be collected in association with the trial from the moment they inform the research team.

### Monitoring

The inclusion of each individual patient in the study is indicated in the electronic hospital information system, and this is, thus, visible to all physicians and nurses involved in the care of the patient. This facilitates the reporting of adverse events to the principal investigator. The principal investigator will report suspected unexpected serious adverse reactions to the federal health authorities.

An external independent CRO will monitor the trial. It will verify the correct completion of data files, correct manipulation of the samples, compliance with local data protection laws, and personal confidentiality. The CRO will follow up the study each week from the beginning of recruitment until the last patient is enrolled. An inspector will be allowed to visit all the center’s facilities to evaluate the data as well as the quality and integrity of the study. At the center, the study files will be reviewed and compared directly with the source documents. The inspector will also discuss the development of the study with the researcher and will verify that the facilities are still acceptable. Only two members of the research group will have access to the identification codes corresponding to the personal data of the patients included in the trial. All important protocol modifications will be communicated to AEMPS (Spanish health authorities).

### Access to data and dissemination policy

The study data will be kept in a locked cupboard, and only members of the research team and the external CRO will be able to access the database. The results of the study will be made public through publication in scientific journals and conferences related to anesthesia in thoracic surgery, as well as through the platform of ClinicalTrials.org.

### Power calculation

The primary outcome of this clinical trial is the comparison of the proportion of patients included in the different C–D classifications, which indicate the severity of postoperative complications for thoracic surgery. Two comparisons are planned: IV lidocaine + SS-PV vs remifentanyl IV+ SS-PV, and lidocaine PV + SS-IV vs remifentanyl IV+ SS-PV. The sample size is calculated based on the C–D classification ordinal scale. According to a similar study design by our research group, the standard deviation of this scale is approximately 1.5 units. Thus, it is necessary to include 48 patients per group with an alpha risk of 5% and a beta risk of 10% in a two-tailed comparison to detect differences ≥ 1 unit. Because losses are estimated to be 5%, the final sample size should be at least 153 patients (sample size calculator GRANMO version 7.12 April 2012).

#### Statistical analysis

Descriptive statistics (mean (standard deviation; SD) or median (IQ25–75)) will be used for continuous variables. Normality will be assessed using the Kolmogorov–Smirnov test. When the variables have a normal distribution, ANOVA and a post hoc Bonferroni test will be applied. If the variables do not have a normal distribution, a non-parametric test will be used to compare them. In this case, the Kruskal–Wallis test and Mann–Whitney test will be used to make post hoc comparisons.

A one-way repeated-measures ANOVA will be performed to analyze repeated measurements of continuous variables. Categorical variables will be presented as absolute frequencies and percentages and compared between groups using the Pearson chi-squared test or Fisher exact test. The odds ratio will be calculated with the 95% confidence interval for categorical postoperative outcome variables. The statistical analyses will be performed using SPSS version 21.0. Statistical significance is set at *p* < 0.05.

Before the digitization and statistical analyses, an investigator will review the data record form for completeness and correctness. At this time, missing data will be identified, drawn from source data, and filled into the case record forms if possible. We do not expect to obtain missing data with outcome variables because they are recorded in a mandatory manner in the electronic hospital files. However, missing pre- or intraoperative data will be treated with a regression imputation method. Data will be analyzed according to the intention-to-treat principle.

## Discussion

The main objective of this study is to compare the clinical outcomes of patients undergoing LRS who are managed using different anesthetic protocols. If patients receiving IV lidocaine have a similar postoperative course (intensity of postoperative pain or perioperative complications) as that of patients receiving PV lidocaine, potential complications that are directly associated with this regional blockade technique could be eliminated (e.g., required time, catheter insertion, intrapleural puncture, bleeding, inadequate subdural or epidural injection).

Two main advantages have been proposed for the intraoperative use of local anesthetics in anesthetic regional blocks. One advantage is the effect of blocking the Na^+^ channels on the nerves and the consequent attenuation of the perioperative neuroendocrine response, and the other advantage is the systemic absorption of the local anesthetic into the blood through administration in the PV space, as well as the consequent anti-inflammatory effects that are associated with LA [13]. Blocking Na^+^ channels using local anesthetics does not seem to be related to inflammation control because these effects occur many hours after lidocaine has been metabolized. Furthermore, it has been observed that intravenous lidocaine administration has systemic analgesic properties [23].

Regarding the precautions for the use of IV lidocaine, at high plasma concentrations (5 μg/mL), toxic and undesirable side effects may appear, including overall cardiac effects and central nervous system effects. We do not expect to obtain such high values based on clinical studies that have used even higher doses than in this study [24, 25]. In addition, the study drug will only be used in the operating room under ECG monitoring, and we will not include patients who have liver disease.

Surgeons and anesthesiologists have used many classifications to perform clinical studies comparing the impact of measurements used in the pre-, intra-, or postoperative period. However, the overall impact on the postoperative course has not been taken into account. Most of the studies that analyze PPCs make a composite variable that comprises all the complications that affect the respiratory tract but have very different severity and impact on the postoperative course. For example, the presence of cough, suspicion of infection, pulmonary edema, and subsegmental atelectasis are usually included as PPCs. However, the clinical relevance of these complications is much lower than that of other PPCs, such as pneumonia, lobar atelectasis, or adult respiratory acute syndrome (ARDS). Thus, it is not surprising that there are studies that describe a similar hospital stay between groups despite observing a different incidence of PPCs related to an intervention because certain complications have little effect on the postoperative treatment provided to the patients. This occurs in studies in which all postoperative complications are presented as a single composite variable without taking into account the severity of each of complication. In our research, we want to determine the real impact on the postoperative course of using IV or PV lidocaine. Thus, we will use the C–D classification to rate the postoperative complications and evaluate the intensity of therapeutic measures that are not routinely included within the normal postoperative course.

A limitation of the study is the short-term follow-up of the postoperative inflammatory response. At the pulmonary level, only inflammatory biomarkers will be monitored during surgery, and at the systemic level, this monitoring will be extended up to 24 h postoperatively. The expression of inflammatory mediators varies over time and peaks within hours to days depending on the mediator analyzed. In a previous study, however, we observed a relationship between PPCs and inflammation biomarkers in the same period of time [2]. Another potential limitation of the study is related to the PV administration of ropivacaine at the end of surgery. Patients in the two groups who receive PV saline during surgery will have a lower effective concentration of ropivacaine in the PV space by dilution of local anesthetic in PV space. This suggests a bias for the assessment of analgesia in the first measure performed in the PACU, but we do not think that it will affect measurements taken during the rest of the study.

In this study, we intend to elucidate the role of lidocaine when it is administered using IV or PV routes in the potential attenuation of the systemic inflammatory response, postoperative pain, and other postoperative courses. In addition, by monitoring the levels of lidocaine in the blood, we can estimate whether there is a dose-dependent relationship with the biomarkers of inflammation or other prognostic variables Additional file 1.

## Trial status

After we obtained the approval of the local ethics committee we included the first patient in the study on January 29, 2019. We plan to spend 30 months to include 153 patients and complete the trial in June 2021. Protocol number IGGFGG-2016 version 4.0 27th April 2017.

### Additional file

Additional file 1: SPIRIT 2013 Checklist: Recommended items to address in a clinical trial protocol and related documents. (DOC 121 kb)

## Appendix 1

**Table 1:** Clavien-Dindo classification

	Grade	Definition
Minor complications	I	Any complication without need for pharmacologic treatment or other intervention
	II	Any complication that requires pharmacologic treatment or minor intervention only
Major complications	III	Any complication that requires surgical, radiologic, endoscopic intervention, or multitherapy
	IIIa	Intervention does not require general anesthesia
	IIIb	Intervention requires general anesthesia
	IV	Any complication requiring intensive care unit management and life support
	IVa	Single organ dysfunction
	IVb	Multiorgan dysfunction
	V	Any complication leading to the death of the patient

## Appendix 2

**Table 2:** Definition of postoperative pulmonary complications

Complication	Definition
Respiratory infection	When a patient received antibiotics for a suspected respiratory infection and met at least one of the following criteria: new or changed sputum, new or changed lung opacity, fever, leukocyte count > 12,000/μ
Respiratory failure	When postoperative PaO_2_ < 60 mmHg on room air, ratio of PaO_2_ to inspired oxygen fraction < 300 or arterial oxyhemoglobin saturation measured with pulse oximetry < 90% and requiring oxygen therapy
Pleural effusion	Chest X-ray demonstrating blunting of the costophrenic angle, loss of the sharp silhouette of the ipsilateral hemidiaphragm in upright position, evidence of displacement of adjacent anatomical structures, or (in supine position) a hazy opacity on one hemithorax with preserved vascular shadows
Atelectasis	Lung opacification with a shift of the mediastinum, hilum, or hemidiaphragm toward the affected area, and compensatory overinflation in the adjacent nonatelectatic lung
Pneumothorax	Air in the pleural space with no vascular bed surrounding the visceral pleura
Bronchospasm	Newly detected expiratory wheezing treated with bronchodilators
Aspiration pneumonitis	Acute lung injury after the inhalation of regurgitated gastric contents

## Appendix 3

**Table 3:** Spirit figure

Visit	Visit 1 (day − 1)	Visit 2 (day 0)	Visit 3 (day 0)	Visit 4 (day 1)	Visit 5 (day 3)	Visit 6 (discharge)	Visit 7 (30 day)
PLACE	Ward	OR	PACU	Ward/PACU	Ward/PACU	Ward	Medical consulting
Study procedures							
Informed consent	X						
Medical records	X						
Demographic data	X						
Clinical course		X	X	X	X	X	X
Intervention		X					
tests							
Hemogram	X	X	X		X	X	
Gasometry	X	X	X				
Biochemistry	X		X		X	X	
Lidocain blood		X					
Chest X-ray	X		X			X	
ECG	X		X		X		
Mini-mental test	X				X		
Biomarcadores							
BLA		X					
Blood		X	X				
PO events							
Mortality		X	X	X	X	X	X
PO complications			X	X	X	X	X

*OR* operating room, *PACU* postoperative care unit, *BLA* bronchoalveolar lavage, *PO* postoperative

## Abbreviations

AKIN: Acute kidney injury network
APU: Acute pain unit
ARDS: Adult respiratory acute syndrome
BAS: Baseline
Cdyn: Lung dynamic compliance
ECG: Electrocardiography
ERAS: Enhanced recovery after surgery
ICU: Intensive care unit
IL: Interleukin
IV: Intravenous
LA: Local anesthetic
LBA: Bronchoalveolar lavage
LRS: Lung resection surgery
OLV: One-lung ventilation
PACU: Postoperative acute care unit
PCA: Patient controlled analgesia
PEEP: Positive end expiratory pressure
POCD: Postoperative cognitive dysfunction
PPC: Postoperative pulmonary complication
SS: Saline serum
TEA: Thoracic epidural anesthesia
TLV: Two-lung ventilation
TPVB: Thoracic paravertebral block
TV: Tidal volume
VAS: Visual analogic scale
VATS: Video-assisted thoracoscopy
